# The Role of Epidermal Growth Factor Receptor Mutations and Epidermal Growth Factor Receptor-Tyrosine Kinase Inhibitors in the Treatment of Lung Cancer

**DOI:** 10.3390/cancers3022667

**Published:** 2011-06-10

**Authors:** Shih-Chieh Chang, Cheng-Yu Chang, Jin-Yuan Shih

**Affiliations:** 1 Department of Internal Medicine, National Yang-Ming University Hospital, Yilan 260, Taiwan; E-Mail: dtsurga9@yahoo.com.tw (S.-C.C.); 2 Department of Chest Medicine, Far Eastern Memorial Hospital, Taipei 220, Taiwan; E-Mail: koala2716@hotmail.com (C.-Y.-C.); 3 Department of Internal Medicine, National Taiwan University Hospital and College of Medicine, National Taiwan University, Taipei 100, Taiwan

**Keywords:** epidermal growth factor receptor, tyrosine kinase inhibitors, gefitinib, erlotinib

## Abstract

Lung cancer is the leading cause of cancer-related deaths worldwide. Non-small-cell lung cancer (NSCLC) cases comprise approximately 85% of the lung cancer cases. Before the era of target therapy, platinum-based doublet chemotherapy only led to a median survival of 8–9 months and a one-year survival of 30%–40% in patients with advanced NSCLC. In July 2002, gefitinib, a small-molecule epidermal growth factor receptor-tyrosine kinase inhibitor (EGFR-TKI), was approved for the treatment of patients with advanced NSCLC in Japan. After the widespread use of gefitinib in the treatment of NSCLC, there have been many new studies regarding the association between the clinical anticancer efficacy of gefitinib and the somatic EGFR mutation status in patients with NSCLC. This article summarizes the role of EGFR mutations in lung cancer and the use of EGFR antagonists in the treatment of lung cancer and its associated adverse effects.

## 1. Introduction

The epidermal growth factor receptor (EGFR) is a transmembrane receptor of the ErbB family, which consists of the following four closely related members: HER1/ErbB1, HER2/neu/ErbB2, HER3/ErbB3, and HER4/ErbB4 [1,2]. Activation of the EGFR can trigger intracellular signal transduction and subsequent cancer cell proliferation, inhibition of apoptosis, neoangiogenesis, and further metastasis, all of which are important for the development of the cancer phenotype [2]. Mutations in the EGFR may be involved in the pathogenesis of lung adenocarcinoma. In animal models, inducible EGFR mutations in exon 19 deletions and a point mutation in exon 21, leading to arginine for leucine substitution at position 858 (L858R), can lead to progressive lung tumorigenesis from atypical adenomatous hyperplasia to invasive adenocarcinoma [3]. Molecular studies have shown that mutant EGFRs can transform fibroblasts and pulmonary epithelial cells without binding with exogenous epidermal growth factors [4].

## 2. EGFR Mutations in Lung Cancer

EGFR mutations are usually found in the first four exons of the tyrosine kinase domain of the *EGFR* gene. Common mutations are as follows: Substitutions for G719 in the nucleotide-binding loop of exon 18, in-frame deletions in exon 19, in-frame duplications and/or insertions in exon 20, and substitutions for L858 or L861 in the activation loop of exon 21 [5]. More than 80% of the kinase domain mutations in EGFRs involve in-frame deletions in exon 19 or L858R of exon 21 [2]. The frequency of EGFR mutations varies with the ethnicity, sex, smoking status, and histological type of lung cancer. The molecular features of lung cancers in patients with minimal tobacco exposure can be similar to those of lung cancers in nonsmoking patients. Furthermore, the EGFR-mutation rate decreases as the number of pack-years increases [6]. The EGFR status of tumors can be evaluated using three major methods: Immunohistochemical (IHC) analysis (at the protein level), fluorescence *in situ* hybridization (FISH) (at the DNA copy number level), and mutational analysis (at the DNA sequence level). EGFR mutations in squamous cell carcinoma and small-cell lung cancer (SCLC) are very rare and are usually found in less than 3% of cases [7,8]. Lung adenocarcinoma has the highest possibility (10%–40%) of harboring somatic mutations in the ATP-binding kinase domain of EGFR. Several investigations have also revealed that patients with lung adenocarcinoma in Asia (30%–50%) show a higher frequency of EGFR mutations than those in the United States (10%) [2,9,10].

In cases in which the primary tumors show EGFR mutations, the corresponding metastatic tumors may not show EGFR mutations. We analyzed the EGFR mutation status in 67 paired tissues samples (primary and metastatic tumors) using the Scorpion Amplified Refractory Mutation System assay, a 27% of discordant rate was found. Therefore, identification of EGFR mutations in only primary tumors may not be representative of the EGFR mutation status of other metastatic lesions; as a result, tyrosine kinase inhibitor (TKI) treatment may have different effects on primary and metastatic tumors [11]. In addition to lung tumor specimens, pleural effusions containing cancer cells can be easily collected and are also available for the detection of EGFR mutations. Malignant pleural effusions are often seen in patients with adenocarcinoma because of the characteristics of the tumor, which grows in the periphery and easily invades the pleural cavity. The EGFR-mutation rate varies from 9.1% to 68.4%, depending on the methodology, patient selection, geographic differences, and positive results for malignant cells (using cytological examination) [12-14]. In a previous study using RT-PCR and direct sequencing method, patients with malignant pleural effusions related to lung adenocarcinoma had a higher EGFR-mutation rate (68.4% *vs.* 50.5%, *p* = 0.007) than the patients who underwent surgical resection for lung adenocarcinoma without malignant pleural effusion. The EGFR mutation-rate in patients with malignant pleural effusions was not associated with smoking status, sex, age, or cancer stage [15].

In our study, where the EGFR sequencing results of 76 SCLC patients were evaluated, only two patients (2.6%) showed EGFR mutations (exon 19 deletions). One patient received gefitinib as salvage therapy but showed no treatment effects [7].

## 3. EGFR Antagonists in the Treatment of Lung Cancer

After two decades of advances in pharmacological development, several EGFR-targeting drugs have been applied in the treatment of non-small-cell lung cancer (NSCLC). They comprise small-molecule TKIs such as gefitinib, erlotinib, monoclonal antibodies, and cetuximab.

### 3.1. EGFR Mutations and EGFR-TKI Efficacy

The current knowledge on the relationship between EGFR mutation status and small-molecule TKI treatment response has resulted in an obvious improvement in the treatment of NSCLC. Gefitinib is used as an effective agent for the treatment of NSCLC, especially in certain patient subgroups, such as women, Asian patients, patients with adenocarcinoma, nonsmokers, and patients with specific EGFR mutations [16,17]. As an initial treatment for pulmonary adenocarcinoma among nonsmokers or former light smokers in East Asia, gefitinib is superior to carboplatin plus paclitaxel, with respect to progression-free survival in the intention-to-treat population (hazard ratio for progression or death, 0.74; 95% confidence interval, 0.65–0.85; *p* < 0.001) [18]. In comparison with docetaxel, gefitinib therapy offers comparable clinical efficacy and a better quality of life when used as second-line treatment in previously treated NSCLC patients [19]. In a previous study of EGFR-TKI treatment in chemonaive patients with specific EGFR mutations, such as exon 19 deletions and substitutions at L858R, the treatment effect of EGFR-TKIs was sustained for up to 8–9 months and was significantly superior to the treatment effect of platinum-based chemotherapy [17]. In the Phase III randomized control trial of gefitinib *vs.* carboplatin/paclitaxel in Asia (IPASS) [18], gefitinib treatment led to significantly longer progression-free survival (PFS) than carboplatin-paclitaxel treatment among the patients who had pulmonary adenocarcinoma with EGFR mutations (hazard ratio for progression or death: 0.48; 95% confidence interval: 0.36–0.64; *p* < 0.001). In a subgroup of 176 patients who showed negative results for mutation, PFS in the patients who received gefitinib was significantly shorter than in those who received carboplatin/paclitaxel (hazard ratio for progression or death with gefitinib: 2.85; 95% confidence interval: 2.05–3.98; *p* < 0.001). In a cohort of 230 chemonaive Japanese patients with metastatic NSCLC and EGFR mutations, the patients who received gefitinib as first-line treatment showed a significantly higher response rate (73.7% *vs.* 30.7%, *p* < 0.001) and median PFS (10.8 months *vs.* 5.4 months, *p* < 0.001) than those who received carboplatin/paclitaxel [20].

Both gefitinib (among never-smokers and patients of Asian origin) and erlotinib, when used as second-line treatments, offer survival benefits for previously treated patients with advanced NSCLC [16,21]. In a previous study of 50 patients who received gefitinib for advanced NSCLC after first-line chemotherapy, the EGFR mutation status, rather than the previous chemotherapy regimens, influenced the effectiveness of gefitinib as a second-line treatment. The second-line gefitinib response rates in patients with EGFR mutations and those with wild-type EGFRs were 71.4% and 13.6%, respectively [22].

The influence of gefitinib administration timing in the treatment of patients with NSCLC has been investigated. In a previous study, 152 patients with exon 19 deletions or L858R mutations were analyzed. Gefitinib treatment resulted in a higher response rate (75.8 % *vs.* 54.1%, *p* = 0.005) in the chemo-naive group (n = 91) than the chemotherapy-treated group (n = 61), but there was no difference between the overall survival of the two groups (16.9 *vs.* 14.7 months, *p* = 0.207) [23].

Even after the failure of first-line gefitinib therapy in patients with advanced NSCLC, subsequent administration of erlotinib as a second-line treatment showed a response rate of 5.6% [24]. Therefore, erlotinib may be used as a salvage treatment when treatment with cisplatin-based regimen and gefitinib fails. Cetuximab is a monoclonal antibody targeting the extracellular domain of EGFR. Patients who underwent cetuximab monotherapy after EGFR-TKI treatment showed no clinical response [25]. In our experience, cetuximab-containing chemotherapy might add a benefit in treatment after failure of gefitinib [26]. Treatment with cetuximab should be investigated further, even in those patients who have previously received EGFR-TKI treatment.

### 3.2. EGFR Mutations and Efficacy of Chemotherapy

In East Asian NSCLC patients with mutated EGFRs, the response rate was higher but not statistically significant, in comparison with the response rate of patients with wild-type EGFRs who underwent first-line chemotherapy (44.6% *vs.* 30.6%, *p* = 0.162). In the analyses of progression-free and overall survival, EGFR gene mutation is not a predictive biomarker of cytotoxic chemotherapy [27]. A similar result was observed in the Iressa Pan-Asia study (IPASS), where the PFS was not different between the patients with or without mutations who received carboplatin/paclitaxel treatment as first-line treatment. However, EGFR mutations seemed to have a beneficial influence on the treatment response of pemetrexed in patients with adenocarcinoma. In a previous study of 156 lung adenocarcinoma patients with measurable target lesions and EGFR sequencing results, the patients with EGFR mutations (N = 93) had a better response rate (12.9% *vs.* 1.6%, *p* = 0.016) and longer PFS (3.9 months *vs*. 2.3 months, *p* = 0.030) than those with wild-type EGFRs (N = 63) after receiving pemetrexed treatment [28].

### 3.3. Innate and Acquired Resistance to EGFR-TKI

Not all EGFR gene mutations in the regions that encode tyrosine kinase are associated with effective responsiveness to gefitinib. EGFR exon 20 mutations are associated with poor gefitinib treatment response. An objective response rate of 25% for gefitinib treatment was reported in 16 patients with exon 20 mutations, and it was much lower than the response rate of patients with deletions in exon 19 and L858R mutations. Furthermore, three of the four responders with exon 20 mutations also had concurrent sensitizing EGFR mutations (L858R) [29]. According to previous studies, the average response rate for gefitinib treatment in patients with exon 20 mutations was only 34%. These mutations in exon 20 could be point mutations, such as V765M, S768I, H773R, and T790M. The mutations also include in-frame duplications and/or insertions, such as A767_V769dupASV or H773_V774insH. These resistant exon 20 mutations could be innate or acquired after the patients had received gefitinib treatment. However, further large prospective studies with a sufficient case number are required. In addition to mutations in exon 20, *MET* oncogene amplification is another possible mechanism of acquired resistance to TKIs. In a previous study of 95 EGFR mutant tumors from untreated patients and patients with acquired resistance, *MET* amplification was found in nine of 43 (21%) patients with acquired resistance, in comparison with the incidence of this amplification in untreated patients (2 of 62; 3%; *p* = 0.007) [30]. Drug resistance and tumor relapse ultimately develop even in NSCLC patients with EGFR mutations, who show a very good response to EGFR-TKIs.

BIBW 2992, an irreversible dual inhibitor of EGFR and HER2 kinase, has shown its efficacy in NSCLC harboring EGFR activating mutations in several preliminary studies. It is also currently being investigated in the management of NSCLC patients failing erlotinib and gefitinib [31,32].

### 3.4. EGFR-TKI Treatment in Patients with Brain Metastasis

In comparison with other solid tumors, lung cancer often progresses to intracranial metastasis and leads to neurologic deficit. For patients with symptomatic brain metastasis, chemotherapy alone usually has poor efficacy in intracranial disease control; whole-brain radiation therapy has proven to be beneficial in prolonging the survival of these patients. In patients with lung adenocarcinoma and brain metastasis, the EGFR mutation status and concurrent administration of EGFR-TKIs were independently associated with the response to whole-brain radiation therapy and better survival [33]. Kim *et al.* [34] reported that EGFR-TKI treatment had an intracranial tumor response rate of 73.9% in nonsmokers with adenocarcinoma of the lung and asymptomatic synchronous brain metastasis. Therefore, EGFR-TKIs may play an important role in the management of lung adenocarcinoma with brain metastasis [35,36]. However, whole-brain irradiation or stereotactic radiosurgery may be needed if disease progression is observed.

### 3.5. EGFR-TKI Treatment in Octogenarians and Patients with Poor Performance Status

For octogenarians with advanced NSCLC, chemotherapy often induces significant adverse effects, and some physicians only offer supportive care. In a retrospective study of 203 patients aged 80 years or older and with advanced NSCLC, the patients who received EGFR-TKI therapy showed better overall survival than those who received only supportive care (median survival: 222 *vs.* 58 days, hazard ratio: 0.54, 95% confidence interval: 0.38–0.76, *p* = 0.0005) [37].

The best supportive care is usually recommended for the NSCLC patients with poor performance status [Eastern Clinical Oncology Group (ECOG) 3 or 4]. In a multicenter Phase II study, 30 EGFR mutation-positive patients with advanced NSCLC showed extremely poor performance status at the start of gefitinib administration as first-line treatment. The overall response rate was 66% and progression-free survival was 6.5 months. Although the response rate was relatively lower in the EGFR-mutant patients with poor performance status, gefitinib may offer survival benefits in some cases [38]. Lee *et al.* [39] also reported that first-line gefitinib treatment may provide clinical benefits for patients with NSCLC and poor performance status, especially in nonsmoking female patients with adenocarcinoma (response rate: 50%, median PFS: 130 days, median overall survival: 236 days).

### 3.6. Combination of EGFR-TKI Treatment and Cytotoxic Chemotherapy

In comparison with the use of only chemotherapy, the concurrent use of gefitinib and erlotinib with platinum-based doublet chemotherapy did not increase survival benefits in patients [40-43]. A randomized, placebo-controlled, Phase II study of sequential erlotinib administration following platinum-based chemotherapy as first-line treatment improved the PFS (29.4 *vs.* 23.4 weeks, hazard ratio: 0.47, 95% confidence interval: 0.33–0.68, *p* = 0.002) of the patients, in comparison with the PFS of patients who underwent only chemotherapy. However, there was no significant difference between the overall survival of the two groups [44].

A randomized Phase II study also revealed erlotinib alone, or in combination with carboplatin/paclitaxel in never or light former smokers with advanced lung adenocarcinoma, had similar progression-free survival (6.7 months *vs.* 6.0 months) [45].

## 4. Adverse Effects of EGFR Antagonists

### 4.1. Skin Rash and Diarrhea

Gefitinib and erlotinib are well tolerated, and the common adverse effects such as skin rash and diarrhea are mild in severity and manageable [2]. The skin rashes caused by EGFR-TKIs are dose-dependent, and reduction of the dosage usually ameliorates the rashes. In genotyping analysis, EGFR intron 1 dinucleotide repeat polymorphism was associated with the occurrence of skin rash in patients who received gefitinib treatment [46].

### 4.2. Interstitial Lung Disease (ILD)

In comparison with platinum-based chemotherapy, gefitinib has modest adverse effects and provides better quality of life [18]. However, ILD is a rare but potentially life-threatening pulmonary toxicity during gefitinib administration [47,48]. The incidence of ILD during gefitinib treatment varies among different ethnicities; up to 2%–4% in the Japanese and 1% worldwide [49]. The main manifestations of gefitinib-induced ILD include dyspnea, nonproductive cough, and profound hypoxemia [50]. Accurate diagnosis is usually difficult since the main pathohistological finding of gefitinib-induced interstitial pneumonia is non-specific diffuse alveolar damage. Erlotinib was also reported to have the potential to induce ILD [51], but the incidence remained uncertain.

The precise mechanism of the interstitial pneumonitis induced by gefitinib or erlotinib remains unknown, although animal models show reduction in EGFR phosphorylation; therefore, suppression of epithelial regeneration may play a role [52]. Cetuximab, a recombinant monoclonal antibody against human EGFR, also inhibits EGFR tyrosine kinase activation, but pulmonary toxicity associated with cetuximab administration is extremely rare [53].

### 4.3. Hepatotoxicity

In previous studies, most patients treated with gefitinib tolerated the drug quite well. Toxicity of the liver has not been commonly reported. However, asymptomatic increase in the levels of liver transaminases has been observed. The Food and Drug Administration (FDA) suggested periodic liver function tests during treatment with gefitinib [54]. Yoshimoto *et al.* [55] reported that 12.2% of the patients had liver injury after gefitinib treatment. This was the first reported incidence of gefitinib-related liver injury. Although, about 90% of gefitinib was recovered in the feces of the patients, surprisingly, in patients with moderate and severely elevated liver tests, the pharmacokinetics was not altered [56]. The cause of gefitinib-related liver damage is still unclear.

## 5. Conclusions

In conclusion, EGFR antagonists play a very important role in the treatment of NSCLC, especially in patients with sensitive EGFR mutations, regardless of chemonaive or previously treated status. For patients with NSCLC and wild-type EGFRs, platinum-based chemotherapy should be considered first, rather than EGFR-TKI treatment. Therefore, measurement of the EGFR mutation status is encouraged for planning an appropriate individualized therapy for patients. Except for ILD, the most adverse effects of EGFR-TKIs are skin rashes and diarrhea, which can be tolerated and are manageable.
